# Role of biochar in anaerobic microbiome enrichment and methane production enhancement during olive mill wastewater biomethanization

**DOI:** 10.3389/fbioe.2022.1100533

**Published:** 2023-01-04

**Authors:** Nozha Abid, Fatma Karray, Imen Kallel, Mariam Slim, Abdellatif Barakat, Najla Mhiri, Mohamed Chamkha, Sami Sayadi

**Affiliations:** ^1^ Laboratory of Environmental Bioprocesses, Center of Biotechnology of Sfax, Sfax, Tunisia; ^2^ Research Laboratory of Environmental Toxicology-Microbiology and Health (LR17ES06), Faculty of Sciences, University of Sfax, Sfax, Tunisia; ^3^ IATE, Montpellier University, INRAE, Agro Institut, Montpellier, France; ^4^ Mohammed VI Polytechnic University (UM6P), Ben Guerir, Morocco; ^5^ Biotechnology Program, Center for Sustainable Development, College of Arts and Sciences, Qatar University, Doha, Qatar

**Keywords:** biochar, direct interspecies electron transfer, anaerobic digestion, prokaryotic communities, olive mill wastewater

## Abstract

The current research work attempted to investigate, for the first time, the impact of biochar addition, on anaerobic digestion of olive mill wastewater with different initial chemical oxygen demand loads in batch cultures (10 g/L, 15 g/L, and 20 g/L). Methane yields were compared by applying one-way analysis of variance (ANOVA) followed by post-hoc Tukey’s analysis. The results demonstrated that adding at 5 g/L biochar to olive mill wastewater with an initial chemical oxygen demand load of 20 g/L increased methane yield by 97.8% and mitigated volatile fatty acid accumulation compared to the control batch. According to the results of microbial community succession revealed by the Illumina amplicon sequencing, biochar supplementation significantly increased diversity of the microbial community and improved the abundance of potential genera involved in direct interspecies electron transfer, including *Methanothrix* and *Methanosarcina*. Consequently, biochar can be a promising alternative in terms of the recovery of metabolic activity during anaerobic digestion of olive mill wastewater at a large scale.

## 1 Introduction

Cultivation of olive trees and oil production are vital activities, mainly in Mediterranean countries. However, uncontrolled olive mill wastewater (OMW) discharge into the environment may beget serious problems owing to its high pollution degree, acidic pH and polyphenols compounds generating antimicrobial effects which involve the inhibition of natural biodegradability of organic load in natural water bodies (Sayadi et al., 2000). Among the numerous processes proposed for the effluent detoxification, biological treatments were considered less expensive and environmental friendly (McNamara et al., 2008). It has been reported that anaerobic digestion (AD) is more efficient than aerobic processes referring basically to the plausibility to treat effluents with high organic load and energy potential like OMW (Gelegenis et al., 2007). In addition, it displays several merits like operational economy, reduction in energy consumption, generation of biogaz and use of the stabilized digestate as a fertilizer (Dareioti et al., 2009).

However, AD effectiveness is limited by the slow metabolism between syntrophs and archaea (Zhao et al., 2016). In fact, some reactor operational modifications may cause volatile fatty acids (VFAs) or hydrogen (H_2_) accumulation that might be toxic to methanogens and acetogenic bacteria respectively, which triggers souring of anaerobic reactors and leads as a matter of fact to the process failure.

Particularly, for OMW, AD was affected chiefly by inhibitory substances such as phenolic compounds as well as long chain fatty acids (Beccari et al., 1999) as they inhibit anaerobic microorganisms. For these reasons, many researchers have been particularly oriented towards improving OMW AD efficiency through dilution, physico-chemical pretreatments, co-digestion or integrated treatments (Khoufi et al., 2015; Vavouraki et al., 2020)*.* Khoufi et al. (2007) proved that OMW pretreatment with electrocoagulation followed by sedimentation led to removal of 76.2% of phenolic compounds and a chemical oxygen demand COD reduction of 43%. After this pretreatment, anaerobic biomethanization was conducted with high methane yield at a loading rate of 6 g COD L^−1^ day^−1^ compared to raw OMW which was toxic to microorganisms. The working mode for syntrophic metabolism during anaerobic methanogenesis was commonly reported as interspecies hydrogen transfer (IHT) (Boone et al., 1999), where H_2_ acts as a diffusive electron shuttle to mediate electron transfer from secondary fermenting bacteria to methanogens. However, the production of hydrogen catalysed by secondary fermenting bacteria is thermodynamically feasible (i.e., ΔG < 0) uniquely if hydrogen concentrations are quite low (H_2_ < 10^–4^ atm (Logan et al., 2002)). Since this condition is accomplished through the consumption of hydrogen by hydrogenotrophic methanogens, the syntrophic metabolism between oxidizing bacteria and archaea is crucial (Mcinerney et al., 2010). Yet, H_2_ diffusion between H_2_ producers and H_2_-consuming methanogens is slow (Stams et al., 2006), which reduces the methane formation rate during AD. Therefore, this syntrophic metabolism network has been confirmed to be metabolically low-efficient.

Over the last years, a new electron transfer pathway, referred to direct interspecies electron transfer (DIET), has been set forward as an alternative network which is more efficient than IHT (Xu et al., 2019). DIET may occur through biological electrical connections such as pili and outer surface c-type cytochromes (Xu et al., 2019). Moreover, several electrically conductive materials, such as graphene (Zhang and Tremblay, 2020) and carbon cloth (Zhao et al., 2016), served as additives for DIET enhancement between syntrophic microorganisms. However, excessive costs as well as the environmental risks of these materials like graphene might limit their use (Kang et al., 2007). Thus, recently, various researchers have investigated biochar as an effective additive to enhance AD. Biochar is an amorphous and a porous carbon-rich material produced by pyrolysis of biomass varieties in the absence or presence of a little amount of oxygen (Cantrell et al., 2012). It is reported that during AD, biochar increases buffering capacity (Zhang et al., 2014), immobilizes microbial cell and improves the methane production rate (Cai et al., 2016). Recently, Wang et al. (2020) have asserted that biochar derived from biowaste promoted DIET to accelerate syntrophic phenol oxidation during AD. The authors suggested a probable shift of syntrophic phenol metabolism from indirect transfer *via* H_2_ to direct interspecies electron transfer.

This work corresponds to a pioneering research that focuses on the impact of the biochar supplementation on AD of OMW. The chief objective of this research work was: Firstly, to examine the impact of different biochar concentrations on biochemical methane potential assays of OMW at different increasing COD loads: 10 g/L, 15 g/L and 20 g/L. Secondly, the prokaryotic community structure, both in the suspended solution and those integrated with biochar surface, were investigated by Illumina to explore their potential implication in DIET.

This work would provide new findings on the effect of biochar supplementation upon the production of methane from OMW which has an important implication for potential application at large scales.

## 2 Materials and methods

### 2.1 Substrates and inoculum

Raw OMW invested in this study was produced by a continuous olive oil mill situated in Sfax (Tunisia). In order to separate suspended solids before use, the samples of OMW were centrifuged by Universal 320 R at 6,000 rpm for10 min. The microbial inoculum was supplied by a semi-pilot anaerobic bioreactor treating OMW and operating in a mesophilic regime.

The used biochar was derived from olive mill wastewater sludge from evaporation ponds in Sfax (Tunisia) and was produced *via* pyrolysis at a temperature of 450 °C. The parameters of the pyrolysis were recently described by Abid et al. (2022). The physico-chemical characteristics of the biochar were: pH = 10.8 ± 0.05; EC = 11 mS/cm; BET surface area (m^2^/g) = 2.77; element contents (oven dry basis): C = 45% +/2.8; N = 2.45% ± 0.13; H = 2.26% ± 0.05; S = 1.23% ± 0.32; O = 19.47% ± 0.3.

### 2.2 Experimental design

The study centered around the impact of the biochar concentration on the AD of OMW at different COD concentrations: 10 g/L, 15 g/L and 20 g/L. Based on previous studies (Luo et al., 2014; Altamirano-Corona et al., 2021), two biochar concentrations were used (5 g/L and 10 g/L).

Batch anaerobic digestion tests were performed in 100 ml batches with a working volume of 60 ml. The inoculum was mixed with a substrate, keeping a volatile solids (VS) ratio (VS substrate to VS inocula) at 1:1(Khoufi et al., 2015). Batches containing inoculum were conducted as blanks to subtract the background gas production. The pH of each batch was adjusted to approximately pH = 7.2 with NaOH (5 M) or HCl (5 M) after which nitrogen gaz was used to purge the system for 3 min so as to remove excess of O_2_ and thus ensure anaerobic conditions. Subsequently, the batches were placed in mesophilic conditions at 37°C.

The volume of methane is measured with a gas trapping device. This device consists of a syringe inserted into the batch through the septum and connected by a flexible tube, to an inverted vial containing a solution of NaOH (3 M) to fix CO_2_.

The tests were set up in triplicate and conducted during an incubation period. The mean values of methane production were calculated. The methane yield was expressed as mL CH_4_/g COD _introduced_ and computed through dividing the cumulative volume of methane produced by the mass of COD introduced into the batch at the start–up. At the end of the incubations, the digestates were separated from the biochars for subsequent analyzes.

### 2.3 Analytical methods

Characterization of OMW as well as the digestates included the following parameters: Total solids content (TS), VS., electrical conductivity (EC), pH, biological oxygen demand (BOD_5_), COD, total polyphenols, and VFAs. pH and EC were measured respectively with a pH meter type Néo Met/Ph- 220 L and a conductivimeter type WTW. TS was measured after oven drying at 105°C by weighing the sample before and after. Afterwards, the retained residues were dried at 105°C. VS. were analyzed by loss on ignition at 550°C for 2 h. COD was determined referring the standard procedure following the American Public Health Association (APHA, 2012). BOD_5_ was specified using the manometric method. Total polyphenols were determined using the Folin-Ciocalteu assay, as reported by Aliakbarian et al. (2015). VFAs were measured by HPLC according to the protocol described by Mechichi and Sayadi (2005).

The physico-chemical characteristics of OMW were pH = 4.9; EC = 14.7 mS/cm; TS = 42.6± 0.46 g/L; VS = 30.3± 0.5 g/L; COD = 47.4± 2.8 g/L; BOD_5_ = 1.75± 0.07 g/L; total polyphenols = 3.4± 0.12 g/L.

### 2.4 Sampling and DNA extraction

Sampling of microbes in the batches was undertaken at the end of methanization according to the method used by (Luo et al., 2014). To investigate the prokaryotic communities in the bottles, three fractions were distinguished as follows: suspended solution, loosely combined with biochar and tightly integrated with biochar surface. Total DNA from all fractions were extracted using DNeasy Power Soil Kit (QIAGEN).

### 2.5 16 S rRNA sequencing analysis

The mixtures of 16 S rRNA gene amplicons (bTEFAP^®^) were generated through the use of a 515F/806R primer set, as previously reported by Dowd et al. (2008) and were sequenced with the MiSeq Illumina (paired-end 2 × 150 bp) platform of the Molecular Research Laboratory (Texas, USA). QIIME 1.9.1 was used to analyze raw data as described by Caporaso et al. (2010). In short, the raw reads were checked for adapter, chimera and low quality sequences. The trimmed reads were clustered into operational taxonomic units (OTUs) using a 97% sequence identity threshold with UCLUST (Edgar, 2010). The Green genes 13.8 database was used to perform taxonomic assignments. Relative abundance of archeal genus were calculated from all archeal sequences. Sequences from all archael OTUs and selected bacterial dominant OTUs (>1% of total sequences) were compared with related sequences retrieved from NCBI databases using BLAST algorithm (Altschul et al., 1990). QIIME software (version 1.9.1) was used to determine the Shannon and Simpson’s diversity indices, the observed species, the Chao1 richness estimator and the phylogenetic diversity index. Venn diagrams were constructed using the VENN DIAGRAM PLOTTER program (http://omics.pnl.gov/software/VennDiagramPlotter.php). The heat map was constructed by the ‘aheatmap’ function in the ‘NMF’ package of R (http://nmf.r-forge.r-project.org/aheatmap.html.

### 2.6 Nucleotide sequence accession numbers

16 S rRNA raw reads from biochar free group (R0) and 5 g/L biochar supplemented group (R1), including suspended fraction of R0 (S0) and R1 (S1), cells loosly (L1) and tightly cells bound to biochar (T1), were deposited in the Short Read Archive of NCBI under project no. PRJNA856838.

### 2.7 Statistical analysis

The experimental values (*n* = 3) were presented in terms of the means ± standard deviation (SD). To determine the significant differences between the cumulative methane productions and methane yields in the batches (*n* = 3), three steps were undertaken. Firstly, the Shapiro-wilk test was used to analyze the normality distribution of variables. Secondly, the one-way ANOVA test was implemented. Thirdly, Tukey’s *post hoc* test was adopted. The significant test was fixed at *p* < 0.05. All statistical analyses were conducted with Statistical Package for the Social Sciences (SPSS) V 20.

## 3 Results

### 3.1 Impact of biochar concentrations on anaerobic digestion of OMW at different increasing COD loads of 10 g/L, 15 g/L and 20 g/L

#### 3.1.1 Impact on the methane production

**FIGURE 1:**
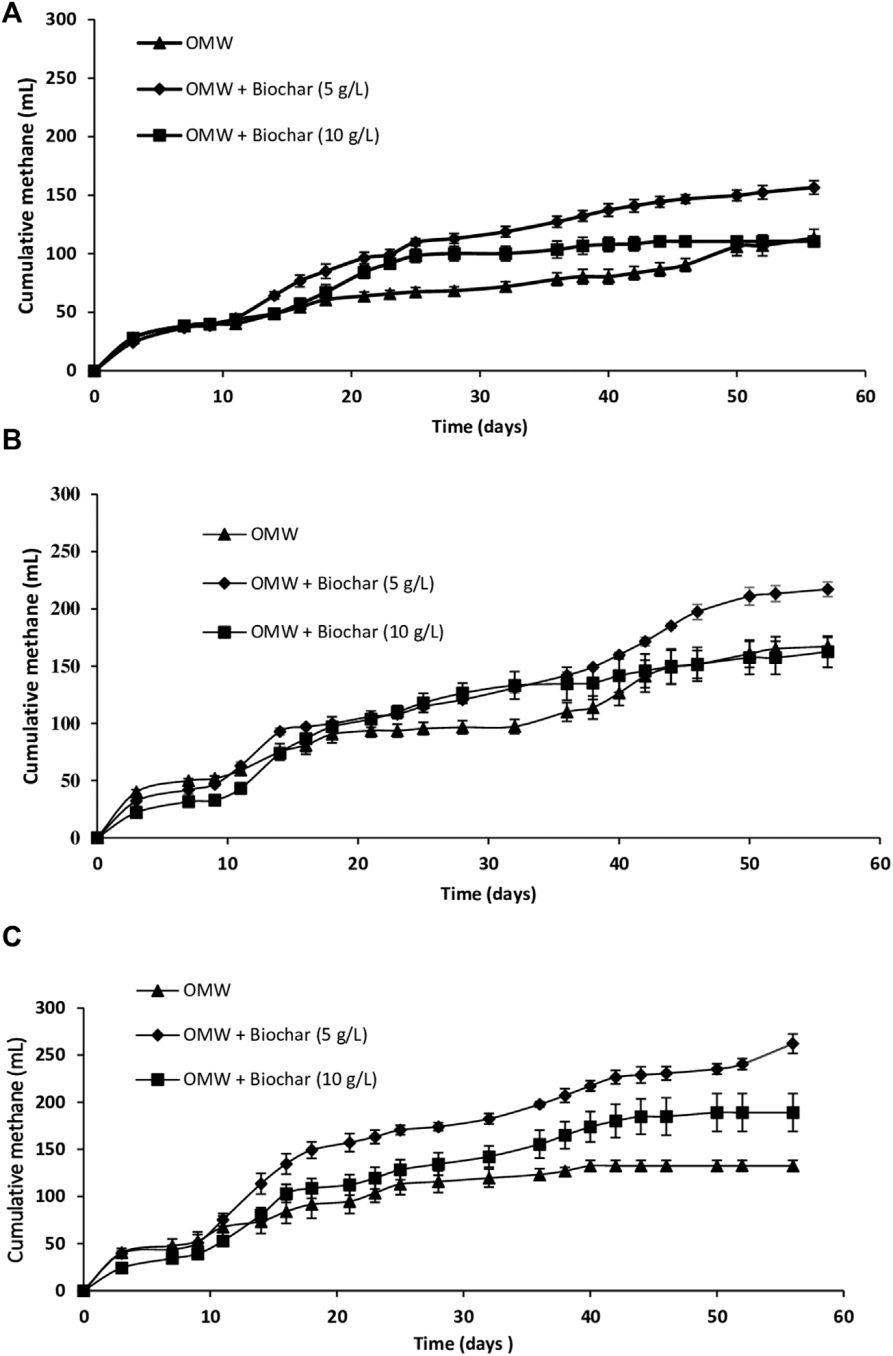
Cumulative methane production during AD of OMW at COD loads of **(A)** 10 g/L **(B)** 15 g/L and **(C)** 20 g/L.

This study purports to assess the influence of biochar concentrations (5 g/L and 10 g/L) on anaerobic digestion of OMW with different increasing COD loads (10 g/L, 15 g/L and 20 g/L) over an incubation period of 56 days. Statistical differences in methane production and methane yields were specified using one-way ANOVA and *post hoc* Tukey’s test analysis with a significance level of 0.05. Figure 1A depicts the cumulative methane production during OMW methanisation at a COD load of 10 g/L.

Over the first 9 days, similar trends in cumulative methane production were observed in all the batches with a low CH_4_ production on day 3 followed by a plateau lasting 6 days which corresponded to the lag time. Subsequently, after 32 days, significant higher cumulative methane production was achieved in batches with 5 g/L and 10 g/L biochar (*p* < 0.05) compared to the control. However, the cumulative CH_4_ production for batches with 5 g/L biochar was significantly higher than that for batches with 10 g/L biochar (*p* < 0.05). At the end, the best significant methane yield (*p < 0.05*) was recorded for the batches with 5 g/L biochar (261.1 ml CH_4_/g COD _introduced_), with a yield improvement of 38.1% compared to the control.

Figure 1B exhibits the cumulative methane production during OMW methanization at a COD load of 15 g/L. As can be inferred, CH_4_ production started after 9 days of lag time in all the batches. Subsequently, the methane production was detected from day 9 to day 18 for the control before it plateaued until day 32; then, it reincreased till the end. This can be assigned to the slow syntrophic degradation during AD of OMW containing slow biodegraded organics (Khoufi et al., 2006). However, on day 32, cumulative CH_4_ productions in cultures with 5 g/L and 10 g/L biochar addition were significantly higher (*p* < 0.05) than the control. However, no significant difference was detected between them (*p* > 0.05). Yield improvements levels compared to the control amounted to 34.6% and 36.9% respectively for batches with 5 g/L and 10 g/L of biochar. From this point onward, a higher increase of methane production was detected in batches with 5 g/L biochar compared to those with 10 g/L till the end of the fermentation. Eventually, after 56 days, a significant higher methane yield was recorded in the batches with 5 g/L biochar (*p* < 0.05) (241.2 ml CH_4_/g COD _introduced_) compared to the control (Table 1) with a yield improvement of 30%.

**TABLE 1:** One-way ANOVA and post hoc Tukey’s test analysis on CH4 yields (mL/g COD introduced) at the end of anaerobic digestion of OMW with initial COD loads of 10 g/L, 15 g/L and 20 g/L.

Treatments	Methane yield (mL/g COD introduced)	Standard deviation	*p* value
OMW_10_	189^ab^	7.2	<0.01
OMW_10_+5g/L Biochar	261.1^d^	5.6	<0.01
OMW_10_+10g/L Biochar	184.5^a^	4.3	<0.01
OMW_15_	185.6^ab^	5.1	<0.01
OMW_15_+5g/L Biochar	241.2^cd^	4.1	<0.01
OMW_15_+10g/L Biochar	180.5^a^	8.7	<0.01
OMW_20_	110.4^e^	2.8	<0.01
OMW_20_+5g/L Biochar	218.4^bc^	4.9	<0.01
OMW_20_+10g/L Biochar	157.6^a^	11.9	<0.01

OMW_10_:OMW with initial load of 10 g/L.

OMW_15:_OMW with initial load of 15 g/L.

OMW_20:_OMW with initial load of 20 g/L.

Different letters reveal a significant difference from post hoc Tukey’s test analysis.

Figure 1C displays cumulative methane production during AD of OMW with 20 g/L of COD load. As demonstrated, after a similar lag time of 9 days, methane production increased in all batches. Afterwards, it plateaued, after 25 and 42 days for the control batch and batch with 10 g/L of biochar, respectively. However, it continued to rise up to 56 days for batch with 5 g/L biochar. After 32 days, biochar supplementation at concentrations of 10 g/L and 5 g/L improved significantly (*p* < 0.05) the methane production compared to the control by 18.6% and 51.9%, respectively. Moreover, cultures with 5 g/L biochar presented significant higher methane production than those with 10 g/L biochar (*p* < 0.05). Likewise, significant yield improvements of 42.7% and 97.8% (*p* < 0.05) were reached at the end of digestion (56 days) for batches with 10 g/L and 5 g/L biochar respectively compared to the control. Consequently, a significant (*p* < 0.05) higher yield (218.4 ml/g COD _introduced_) was achieved in cultures with 5 g/L biochar compared to the control and culture with 10 g/L biochar (Table 1). As expected, a low yield of methane production was recorded during anaerobic digestion in the control batch (110.4 ml CH_4_/g COD _introduced_). This could be assigned to the high COD load and polyphenols contents which entail toxicity leading to a decrease in methane yield (Khoufi et al., 2007).

#### 3.1.2 Variation of pH, VFAs, COD and polyphenols removal

**FIGURE 2:**
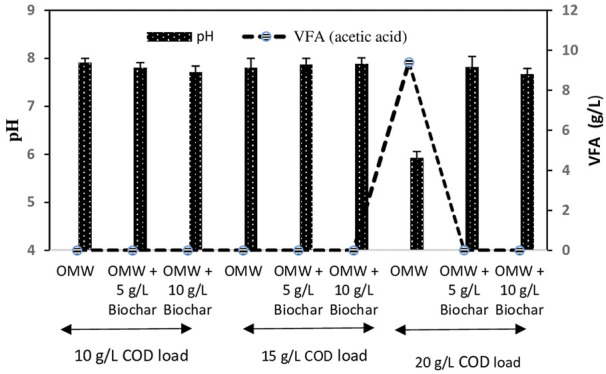
pH and volatile fatty acids concentrations at the end of anaerobic digestion of OMW at different COD loads.

Variation of VFAs has been recognized as key indicators for the unbalance process during AD of OMW (Mechichi and Sayadi, 2005). Final VFAs as well as pH are highlighted in Figure 2. It can be noticed that all treatments except OMW with 20 g/L COD load (control), exhibited suitable pH values between 7.6 and 7.9 and null VFAs accumulation, which reflected the process stability during the methanization. However, in the control cultures with the highest COD load (20 g/L), final acidic pH of 5.92 along with high final accumulation of acetic acid (9.4 g/L) revealed AD inhibition which coincided with the observed lower methane yield of 110.4 ml/g COD _introduced._

**FIGURE 3:**
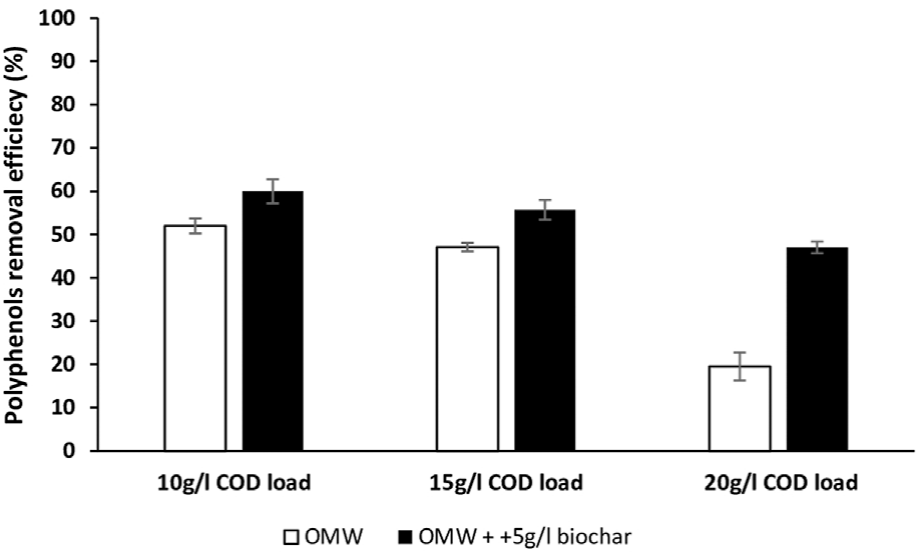
Polyphenols removal efficiencies at the end of anaerobic digestion of OMW at different COD loads.

After 56 days, polyphenols removal ratios were 52%, 47.1% and 19.5% in control cultures with COD loads of 10 g/L, 15 g/L and 20 g/L, respectively. However, biochar supplementation increased polyphenols removal efficiencies by 8, 8.6 and 27.5 percentage points, respectively (Figure 3).

Additionnally, COD removal efficiencies after 56 days in control cultures with COD loads of 10 g/L, 15 g/L and 20 g/L were 55.33%, 54.37% and 32.98%, respectively. Yet, they increased by 21.6, 16.8 and 32.6 percentage points, respectively compared to controls after 5 g/L biochar supplementation. (Supplementary Figure S1).

### 3.2 Response of microbial community structure to biochar supplementation

Microbial communities (bacteria and archaea) were analyzed at the end of operation (56 days) in the cultures with an initial COD load of 20 g/L based on the 16 S rRNA gene amplicon sequencing using high-throughput sequency on an Illumina Miseq platform. Microbial communities of biochar free group (R0) and 5 g/L biochar supplemented group (R1), included suspended fractions of R0 (S0) and R1 (S1), loosly cells (L1) and tightly ones bound to biochar (T1).

**FIGURE 4:**
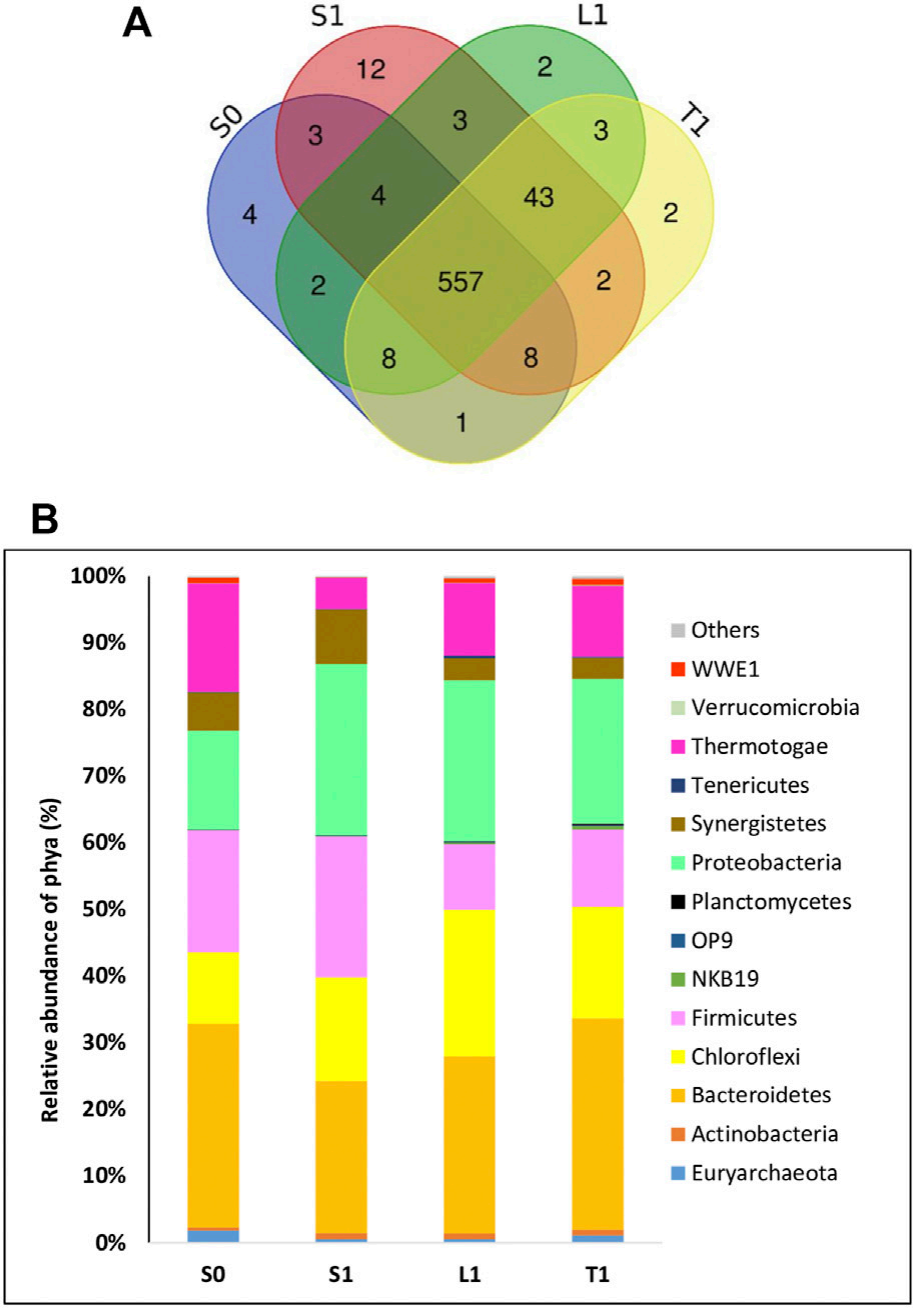
Analysis of microbial communities at the end of AD with initial COD load of 20 g/L in biochar free group (SO) and 5 g/L biochar supplemented group (Sl, Ll, Tl) **(A)** OTUs-VENN diagrams based on high-throughput sequencing analysis **(B)** Major phyla (relative abundance> 1% of all sequences).

As plotted in the venn diagram (Figure 4A), 557 OTUs were shared among all samples.

A high number of unique OTUs was identified in the biochar supplemented samples (S1, L1 and T1) (12 + 2+2 OTUs) comparing to the biochar free group (S0) (4 OTUs).

Chao1, Shannon and Simpson indexes were performed to compare the prokaryotic richness and diversity between the biochar free (S0) and supplemented group (S1, L1 and T1). The diversity and richness estimators obtained from the *Next-Generation Sequencing* (NGS) data, are presented in Table 2. The prokaryotic diversity based on Shannon and Simpson indexes of the S0 group were about 5.258 and 0.936, respectively. However, in the biochar supplemented group (S1, L1 and T1), an increase in Shannon and Simpson indexes was recorded yielding an average of 5.960 and 0.959, respectively. Moreover, the R1 group showed higher Chao1 species richness estimator index (626 average OTUs) compared to the S0 group (587 OTUs). These findings indicated that microbial community structure and diversity changed following biochar addition.

**TABLE 2:** Diversity indexes of microbial community in biochar free group (S0) and biochar supplemented group (S1, L1, T1).

Sample	Seqs/Sample	Chao 1	Observed_OTUs	Shannon	Simpson
S0	223,689	595.724	587	5.258	0.936
S1	215,932	635.000	632	5.999	0.962
L1	196,277	628.429	622	5.954	0.958
T1	203,49	629.000	624	5.928	0.957

The microbial community composition at phylum level is portrayed in Figure 4B. All detected OTUs belong to 14 important phyla whose relative abundance > 0.1%. In the suspended fraction of the biochar free group (S0), the major detected phyla were: Bacteroidetes, Firmicutes, Thermotogae, Proteobacteria, Chloroflexi, Synergistetes and Euryarchaeota accounting for 30.55%, 18.29%, 16.29%, 14.83%, 10.73%, 5.74% and 1.82% respectively. Compared to the suspended fraction, an increase in relative abundance of Proteobacteria and Chloroflexi (14.83% and 10.73% respectively for S0) were recorded in the biochar supplemented groups, amounting respectively to 25.72% and 15.58% in the suspension (S1), 24.15% and 21.98% respectively in lously-bound biomasses (L1) and 21.78% and 16.73% respectively in tightly-bound cells (T1). However, a significant decrease in relative abundance of Thermotogae was noticed in all biomass fractions of biochar supplemented samples (S1 = 4.68%, L1 = 10.91%, T1 = 10.62%) compared to the biochar free groups (S0 = 16.29%).

The members of phyla Bacteroidetes and Firmicutes have important roles during anaerobic digestion in the generation of short chain fatty acids for methane production during hydrolysis and acidogenesis (Pan et al., 2019). Proteobacteria are syntrophic bacteria responsible for the cellulose and protein degradation and are also involved in the degradation of organic acids (Ma et al., 2019). The Chloroflexi phylum is a common fermenting group described in AD reactors. Moreover, Chloroflexi is also known as a phylum of electroactive bacteria (Hoareau et al., 2021). These results suggest that biochar addition improved the rate of hydrolytic as well as electroactive bacteria.

#### 3.2.1 Analysis of bacterial communities

**FIGURE 5:**
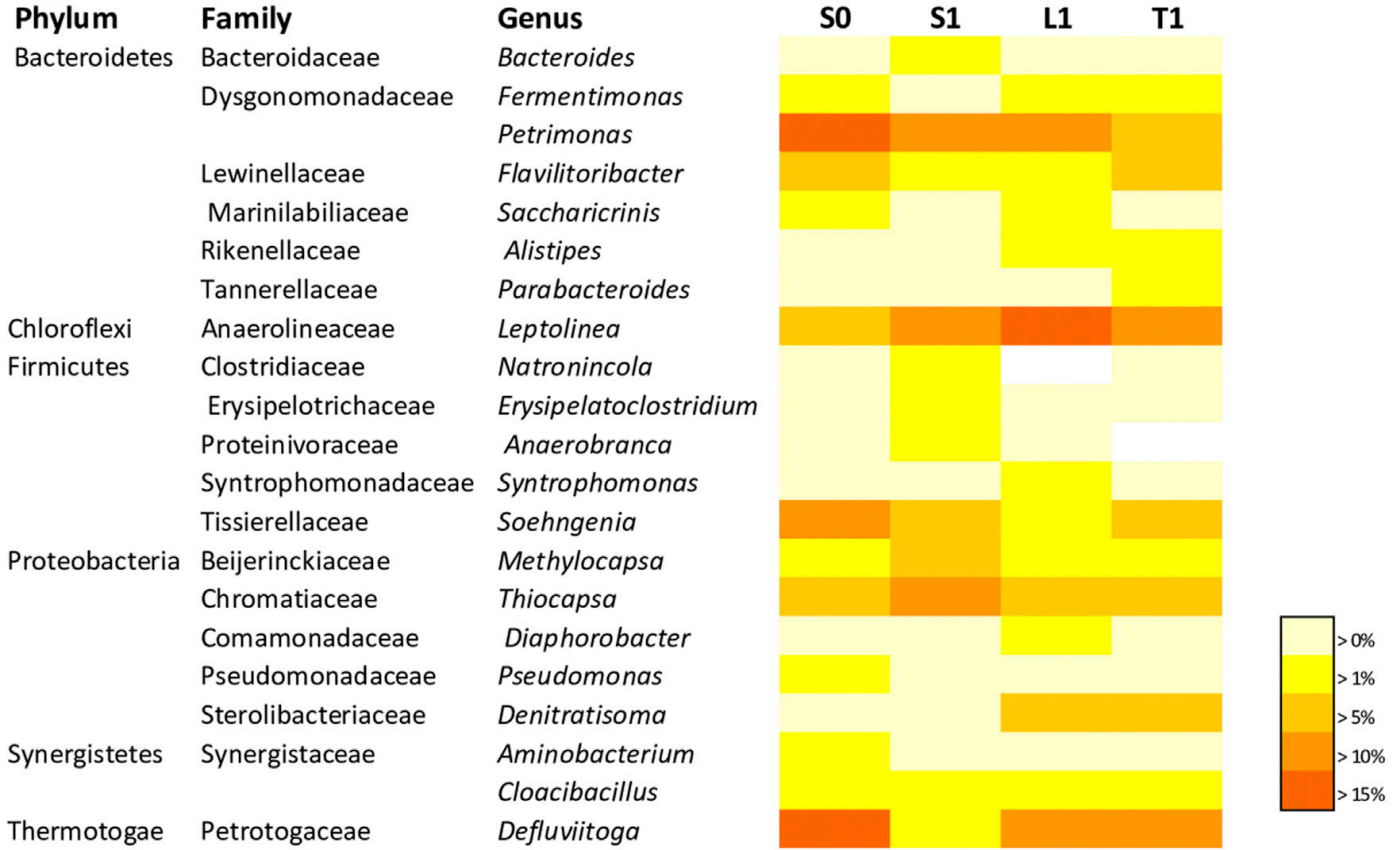
Heat map showing the relative abundance of dominant bacterial genera (> 1% of all sequences) related to the end of AD with initial COD load of 20 g/L in biochar free group. (S0) and 5 g/L biochar supplemented group (S1, L1, T1). The color intensityfor each panel corresponds to the genus abundance; white (0%) indicates low relative abundance, through yellow ( >1%) to red (> 15%) indicate a high level of relative abundance.

As for the bacterial community, taxa displaying a mean proportion of 1% were considered as the most abundant. The taxonomic classification at genus level (Figure 5) disclosed that the most dominant groups in the suspended fraction of the biochar-free group (S0), were *Defluviitoga, Petrimonas, Soehngenia, Leptolinea, Thiocapsa, Flavilitoribacter, Fermentimonas, Methylocapsa, Pseudomonas, Aminobacterium, Cloacibacillus* and *Saccharicrinis* amounting respectively to 16.22%, 15.92%, 11.02%, 9.5%, 9.06%, 5.23%, 2.12%, 1.62%, 1.51%, 1.48%, 1.33% and 1.09% of the sequence reads. The biochar supplemented groups demonstrated changes in the community compositions.

Notably, the genus *Bacteroides* from Bacteroidaceae family tended to be enriched in the suspended fraction in the biochar supplemented reactor (3.37% for S1, 0.27% for L1, 0.65% for T1) compared to the suspended fraction of R0 (S0 = 0.05%). Similar pattern was observed for Clostridiacea with genus *Natronincola,* which was exclusively enriched in the suspended fraction of the biochar supplemented reactor (1.9% for S1, 0% for L1, 0% for T1) while it was absent in the suspended fraction of R0 (S0 = 0%).

Likewise, Erysipelotrichaceae with the genus group of *Erysipelatoclostridium* (*Erysipelatoclostridium ramosum* species) (Supplementary Table S1) were enriched in the suspended fraction of the biochar supplemented reactor (3.48% for S1, 0.17% for L1, 0.13% for T1) compared to the suspended fraction of R0 (S0 = 0.68%).

Proteinivoraceae with the genus of *Anaerobranca* were exclusively enriched in the suspended fraction of the biochar supplemented sample (1.3% for S1, 0% for L1, 0% for T1) while being absent in the suspended fraction of R0 (S0 = 0%). It is well known that it corresponds to be an acidogenic bacteria (Yin et al., 2018). The abundance of *Cloacibacillus* from Synergistaceae family increased by about 3.43 folds in the suspended fraction of the biochar supplemented samples (4.58% for S1, 1.55% for L1, 1.07% for T1) compared to R0 (S0 = 1.33%). Anaerolineaceae with the genus of *Leptolinea* was boosted in the biochar supplemented samples (13.19% for S1, 17.94% for L1, 13.82% for T1) compared to S0 (9.50%). More importanly, Rikenellaceae family with the genus of *Alistipes* was distinctly more detected in the fixed biomass (2.08% for L1, 1.46% for T1) while it was not dominant in the suspended fractions (0.03% for S0 and 0.81% for S1). In this respect, the abundance of *Parabacteroides* from Tannerellaceae family increased exclusively in the loosly fixed biomass by 7.8 folds and in the tightly fixed biomass by 35.6 folds compared to S0 (0.05% for S0, 0.06% for S1, 0.4% for L1 and 1.79% for T1).

As illustrated in Figure 5, heterotrophic denitrifying bacteria were distincly enriched in the fixed biomass. Indeed, the relative percentage of *Denitratisoma* from Sterolibacteriaceae family was higher in the biochar fixed biomass (6.35% for T1, 5.81% for L1) while it was not dominant in the suspended fractions (0.06% for S0 and 0.28% for S1). It seemed that biochar served as a habitat for *Denitratisoma* which are heterotrophic denitrifying bacteria participating in nitrogen removal. Lu et al. (2021) reported that the relative abundance of *Denitratisoma* increased in the granulated activated carbon supplemented up-flow anaerobic sludge blanket reactors compared to the control (without activated carbon). Within this framework, the relative percentage of the genus of *Diaphorobacter* from Comamonadaceae family which is a facultative heterotrophic denitrifier was higher in the biochar fixed biomass and particularly in the loosly–bound fraction (S1 = 0.53%; L1 = 1.3%; T1 = 0.61%) than that in suspended fraction of R0 (S0 = 0.1%). As it has been proven that DIET is also involved in denitrification (Xie, 2006), *Denitratisoma oestradiolicum* and *Diaphorobacter polyhydroxybutyrativorans* (Supplementary Table S1) stand for potential exoelectrogens.

#### 3.2.2 Analysis of archaeal communities

**FIGURE 6:**
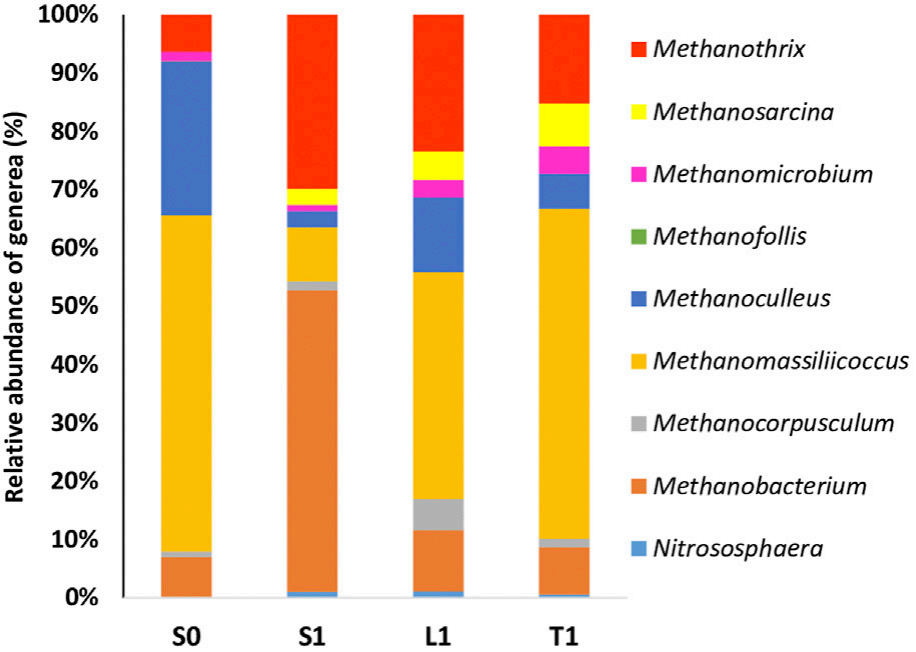
Relative abudance of archaeal community at genus level at the end of AD with initial COD load of 20 g/L in biochar free group (S0) and 5 g/L biochar supplemented group (S1, L1, T1).

As far as the archaeal community is concerned, the taxonomic classification at genus level revealed that the most groups detected in biochar-free group (S0) include *Methanomassiliicoccus, Methanoculleus, Methanobacterium* and *Methanothrix* (Figure 6).

Lower relative abundance of hydrogenetrophic methanogens such as *Methanoculleus* and *Methanomassiliicoccus* in S1 (2.81% and 9.24%, respectively) were detected compared to S0 (26.38% and 57.68%, respectively). However, *Methanobacterium* genus seemed to be enriched in the suspended fraction of biochar treatment (6.83% in S0, 51.76% in S1). A more diverse archaeal diversity was recorded in biochar supplemented groups compared to the control. Basically, *Methanosarcina barkeri* (Supplementary Table S2) was only identified in biochar supplemented groups and was particularly tightly attached to biochar (relative abundance of 7.36% in T1 compared to 4.92% in L1 and 2.72 in S1). Similar pattern was observed with *Methanothrix* which were enriched in biochar treatment samples and particularly in the suspended fraction (relative abundance of 29.84% in S1 compared to 6.26% in S0, 23.38% in L1 and 15.12% in T1).

## 4 Discussion

In the current work, we attempt to assess the influence of biochar concentrations (5 g/L and 10 g/L) on anaerobic digestion of OMW with different increasing COD loads (10 g/L, 15 g/L and 20 g/L) over an incubation period of 56 days. Statistical differences in methane production and methane yields per Gram of COD_introduced_ were determined. When examining the final CH_4_ yield (mL/g COD _introduced_) for all the treatments, ANOVA analysis revealed a significant difference among the various COD loads treatments (Table 1). However, the Tukey’s *post hoc* test highlighted these points: Firstly, a significant lower yield was reached in raw OMW cultures with 20 g/L COD compared to cultures with 10 g/L COD. However, after methane production enhancement with 5 g/L biochar addition, final yield became significantly higher than that achieved in batch with 10 g/L COD load. Hence, we deduce that biochar supplementation during AD of OMW may entail promising results in terms of the recovery of metabolic activity when operating at high organic load rate at large scale applications. Furthemore, for all the treatments, 5 g/L biochar addition improved significantly methane yield compared to the control and treatments with 10 g/L biochar. These results suggested that, for all initial loads of COD, the addition of 5 g/L of biochar was more effective than the 10 g/L dose in increasing yield methane production.

Biochar supplementation may enhance methane production by adsorbing polyphenols which inhibit methanogens. This likelihood may be excluded since a higher concentration of biochar is needed for polyphenols elimination (Abid et al., 2022). It would be highly useful to analyze the microbial communities (bacteria and archaea) at the end of operation (56 days) in the cultures with initial COD load of 20 g/L to get insights into the microbial community response during AD with biochar supplementation. Microbial communities of biochar free group (R0) and 5 g/L biochar supplemented group (R1), involving suspended fraction of R0 (S0) and R1 (S1), loosly cells (L1) and tightly ones bound to biochar (T1) were analyzed. Results from high-throughput sequencing revealed that biochar supplementation improved the abundance of hydrolytic bacteria, mainly in the suspended fraction, such as Bacteroidaceae, as well as acidogenic bacteria such as Clostridiacea, Proteinivoraceae and fermentative genus group such as *Erysipelatoclostridium.* Bacteroidaceae play a key role in organic matter depolymerisation during acidogenesis phase thanks to several hydrolyzing enzymes (Wang et al., 2017). They seemed to be part of bacterial key players during AD exposed to high concentrations of phenol reaching 2 g/L (Poirier et al., 2016). Consequently, they were involved in the hydrolysis step to enhance polyphenols degradation. Our results go in good consistency with those of Pytlak et al. (2020) who asserted that biochar addition to a fermentation sludge containing sugar beet pulp leads to an enrichment of Bacteroidales. On the other side, it was emphasized that *Bacteroides* are probably able for the extracellular electron transfer since some species were enriched on the anode of a bio-electrolysis cell system and were able to transfer electrons directly to ferric iron (Wang et al., 2010). Clostridiaceae were known not only as acidogenic bacteria (Yin et al., 2018) but also as exoelectrogens. In particular, *Natronincola peptidivorans* was reported as an interesting new potential electro-synthesizing bacterium in the Clostridiaceae family capable of direct electron transfer in a microbial electrolysis cell (Quéméner et al., 2019). *Erysipelatoclostridium* were known for their ability to ferment several carbohydrates such as acetate, propionate and butyrate (Yutin and Galperin, 2013).

Stimulated CH_4_ after 5 g/L biochar addition to OMW with initial COD load of 20 g/L may be explained by the bacterial and archaeal communities shift with a significant enrichment of microbes involved in DIET. In this line, our results demonstrated the enrichement of Anaerolineaceae which were known for their capacities for extracellular transfer of electrons using fulvic acids as electron acceptors (Dang et al., 2016). These results proved to be consistent with the findings of Wang et al. (2018) who reported that biochar supplementation during AD of complex organic wastes entailed the enrichment of Anaerolineaceae. In this respect, *Cloacibacillus* species which were enriched in the suspended fraction of biochar supplemented group proved to be syntrophic amino-acid-oxydizing bacteria (Zhao et al., 2017). Recently, Yang et al. (2021) have argued that biochar addition during anaerobic digestion of swine manure enriched *Cloacibacillus* which might participate in DIET with *Methanothrix.*

More importantly*,* our results revealed that Rikenellaceae and *Parabacteroides* were distinctly more enriched in the fixed biomass of biochar. Recently, Rikenellaceae were identified as potential syntrophic bacteria capable of establishing magnetite-mediated direct electron transfer with methanogens to accelerate VFAs degradation (Lee et al., 2019). *Parabacteroides* are fermentative species. However, many researchers have recently reported the abundant growth of *Parabacteroides* under DIET-simulated conditions (Baek et al., 2019), which is suggestive that this group may potentially be involved in electro-syntrophic interactions during anaerobic digestion. Consequently, our results go in good conformity with previous researchers’ findings indicating that these groups may possibly be involved in electro-syntrophic interactions during anaerobic digestion.

Lower relative abundance of hydrogenetrophic methanogens such as *Methanoculleus* and *Methanomassiliicoccus* in S1 compared to S0 proved that IHT wan’t enhanced by biochar. *Methanoculleus* emergence has been reported as an early indicator which portends phenol inhibition towards microbial community during AD exposed to phenol concentrations between 1 and 2 g/L (Poirier et al., 2016). Hence, the rigorous hydrogenotrophic *Methanoculleus* seemed to play a key role to maintain AD *via* IHT in R0. However, *Methanobacterium* seemed to be enriched in the suspended fraction for the biochar treatment. *Methanobacterium* are hydrogenotrophic methanogens, which had been broadly detected in the AD of phenol culture (Na et al., 2016; Poirier et al., 2016). Consequently, positive effects of biochar addition on polyphenols alleviation can be attributed to *Methanobacterium* enrichement. They were recognized also as syntrophic microbes which participated to DIET and proved to be enriched in anaerobic digestion of sewage sludge process with biochar addition (Wu et al., 2019). Recently, they have been reported to be able to change the primary working mode of the syntrophic metabolism from IHT to DIET during anareobic digestion of swine manure with biochar addition (Yang et al., 2021).

**FIGURE 7:**
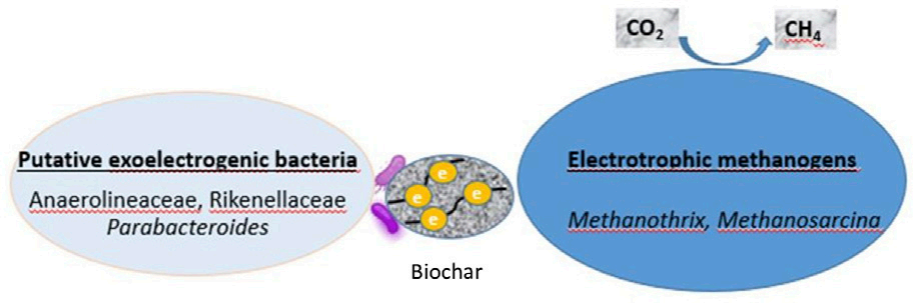
Conceptual illustration mechanism of enhancing anaerobic digestion of OMW by biochar via DIET.

Additionally, the enrichment of genera of archaea like *Methanothrix* and *Methanosarcina* is suggestive that DIET would be accelerated in the biochar treatment samples. *Methanosarcina barkeri* is known as a methanogen that is able to participate to DIET (Rotaru et al., 2014). *Methanothrix* can use both acetoclastic methanogenesis and DIET-CO_2_ reduction during AD with the addition of biochar (Wang C et al., 2018). Thus, the stimulation of methane production rate seems to be associated with the increase in abundance of these genera with independence of the syntrophy between bacteria and archaea *via* IHT. To sum up, several putative exoelectrogenic microbes were enriched in biochar treatment samples and particularly in suspended fractions such as *Bacteroides, Natronincola* and *Cloacibacillus.* More importantly, since the potential exoelectrogenic bacteria like *Parabacteroides* Rikenellaceae and Anaerolineaceae were enriched exclusively on the biochar, they could be directly interacting with archaea of *Methanothrix and Methanosarcina via* DIET. These archaea species which were enriched on the biochar, proved to be responsible for DIET. A Conceptual illustration of DIET possible mechanism in anaerobic digestion of OMW with biochar is described in Figure 7.

As a conductive material, biochar can serve as an electron acceptor as well as a donor and allows DIET to take place (Park et al., 2018). When compared to IHT, DIET has been suggested as faster and energetically more effective than IHT (Xu et al., 2019) since it does not require energy to produce H_2_. As electrical conductivity plays an important role in terms of promoting DIET (Zhao et al., 2017), the used biochar in our study, which was derived from olive mill waste water sludge, would stand for a good candidate as it possesses high electrical conductivity (11 m/cm).

The inhibition of OMW polypenols towards methanogens begets the imbalance in the relationship between bacteria and methanogens, which results in significant VFAs accumulation and AD inhibition in the control cultures with the highest COD load (20 g/L). Furthermore, biochar supplementation at 5 g/L to OMW with initial COD load of 20 g/L mitigated VFAs accumulation. Our findings go in good correlation with the results of Wang G et al. (2018). These authors disclosed that biochar derived from sawdust alleviated VFAs accumulation during AD of complex organic wastes. Positive effects of biochar addition on the VFAs alleviation can be interpreted as follows: Our study revealed that biochar enhanced the growth of bacteria known for their ability to ferment several carbohydrates like acetate, propionate and butyrate (Yutin and Galperin, 2013) such as *Erysipelatoclostridium*. Consequently, enhanced degradation of VFAs was probably ascribed to the improved solubilization of OMW following the bacteria enrichement. Besides, our study demonstrated that biochar addition suppressed IHT and enabled DIET to take place. This pathway enhances the electron transport rates when compared to IHT. Hence, the H_2_ and formate concentrations are lower than during IHT. Therfore, syntrophic VFAs oxydation becomes thermodynamically feasible (Capson-Tojo et al., 2018) *.*

## 5 Conclusion

This study addresses the impact of biochar addition during AD of OMW in batch at different COD increasing loads. AD of OMW with initial COD load of 20 g/L led to process unbalance owing to VFAs accumulation. However, biochar addition at 5 g/L increased methane yield by 97.8%, mitigated VFAs accumulation and enriched prokaryotic as well as methanogenic communities.

Results indicated that biochar displayed a great potential in terms of enhancing VFAs degradation referring to the improved solubilization of OMW through microbial hydrolysis with increased abundance of hydrolytic bacteria such as Bacteroidaceae, as well as acidogenic bacteria such as Clostridiaceae, Proteinivoraceae*.* In addition, the enrichment of genera like *Methanothrix* and *Methanosarcina* may indicate the establishment of DIET in the biochar treatment samples.

This work provides new findings on the effect of biochar supplementation upon the production of methane from OMW which has an important implication for potential applications at large scales.

## Data Availability

The datasets presented in this study can be found in online repositories. The names of the repository/repositories and accession number(s) can be found in the article.
