# Supplementary material for: Role of biochar in anaerobic microbiome enrichment and methane production enhancement during olive mill wastewater biomethanization
Source: Front Bioeng Biotechnol. 2023 Jan 4;10:1100533. doi: 10.3389/fbioe.2022.1100533 (PMC9846136; doi:10.3389/fbioe.2022.1100533)
Supplement: Supplementary file 1 [file Table1.pdf]

Table S1: Phylogenetic affiliation of bacterial OTUs obtained from Illumina Miseq sequencing analysis in control and biochar supplemented batches targeting the 16S rRNA gene

OTU	Sequences per sample (%)				Closet cultivated relative from NCBI nucleotide database		Similarity (%)
	S0	S1	L1	T1	Taxonomy (phylum/family)	Species (accession number)	
753792	5.23	1.48	3.90	9.23	Bacteroidetes ; Lewinellaceae	*Flavilitoribacter nigricans* (NR_115013)	80.22
635500	1.09	0.65	1.21	0.58	Bacteroidetes ; Marinilabiliaceae	*Saccharicrinis aurantiacus* (NR_156071)	86.36
837605	0.03	0.81	2.08	1.46	Bacteroidetes ; Rikenellaceae	*Alistipes communis* (NR_133025)	85.88
572010	0.05	3.37	0.27	0.65	Bacteroidetes ; Bacteroidaceae	*Bacteroides graminisolvens* (NR_113069)	98.87
605125	1.67	1.28	0.72	0.72	Bacteroidetes ; Dysgonomonadaceae	*Petrimonas sulfuriphila* (NR_042987)	94.72
688528	0.16	1.23	1.56	0.92	Bacteroidetes ; Dysgonomonadaceae	*Petrimonas mucosa* (NR_148808)	96.98
256657	1.04	0.36	0.67	0.86	Bacteroidetes ; Dysgonomonadaceae	*Fermentimonas caenicola* (NR_148809)	98.87
110976	7.54	6.48	4.01	4.29	Bacteroidetes ; Dysgonomonadaceae	*Petrimonas sulfuriphila* (NR_042987)	94.34
4323661	1.08	0.36	0.56	0.67	Bacteroidetes ; Dysgonomonadaceae	*Fermentimonas caenicola* (NR_148809)	96.23
547696	6.33	1.22	2.36	1.78	Bacteroidetes ; Dysgonomonadaceae	*Petrimonas mucosa* (NR_148808)	91.32
2532911	0.23	0.83	1.60	1.37	Bacteroidetes ; Dysgonomonadaceae	*Petrimonas sulfuriphila* (NR_042987)	98.87
110066	0.05	0.06	0.40	1.79	Bacteroidetes ; Tannerellaceae	*Parabacteroides distasonis* ATCC 8503 (NR_074376)	82.33
767235	9.03	8.83	9.04	7.23	Chloroflexi ; Anaerolineaceae	*Leptolinea tardivitalis* (NR_040971)	91.22
827364	0.47	4.37	8.90	6.60	Chloroflexi ; Anaerolineaceae	*Leptolinea tardivitalis* (NR_040971)	89.49
261405	9.44	4.32	2.51	3.72	Firmicutes ; Tissierellaceae	*Soehngenia saccharolytica* (NR_117382)	98.86
228603	1.58	1.35	1.02	1.51	Firmicutes ; Tissierellaceae	*Soehngenia saccharolytica* (NR_117382)	98.48
570234	0.00	1.90	0.00	0.00	Firmicutes ; Clostridiaceae;	*Natronincola peptidivorans* (NR_116003)	97.73
539716	0.81	0.38	1.19	0.67	Firmicutes ; Syntrophomonadaceae	*Syntrophomonas bryantii* (NR_104881)	96.23
1075145	0.00	1.29	0.00	0.00	Firmicutes ; Proteinivoraceae	*Anaerobranca gottschalkii* (NR_025050)	93.18
145801	0.68	3.48	0.17	0.13	Firmicutes ; Erysipelotrichaceae	*Erysipelatoclostridium ramosum* (NR_113243)	98.86
701112	0.54	3.29	3.18	0.98	Proteobacteria ; Beijerinckiaceae	*Methylocapsa acidiphila* (NR_028923)	96.98
549898	1.08	2.10	1.17	0.78	Proteobacteria ; Beijerinckiaceae	*Methylocapsa acidiphila* (NR_028923)	97.74
846710	0.10	0.53	1.30	0.61	Proteobacteria ; Comamonadaceae	*Diaphorobacter polyhydroxybutyrativorans* (NR_137222)	98.87
245398	0.06	0.28	5.81	6.35	Proteobacteria ; Sterolibacteriaceae	*Denitratisoma oestradiolicum* (NR_043249)	96.6
821874	1.24	1.99	0.95	1.01	Proteobacteria ; Chromatiaceae	*Thiocapsa imhoffii* (NR_115810)	93.26
160464	7.82	11.45	4.69	6.16	Proteobacteria ; Chromatiaceae	*Thiocapsa imhoffii* (NR_115810)	93.26
735666	1.51	0.00	0.02	0.00	Proteobacteria ; Pseudomonadaceae	*Pseudomonas balearica* (NR_025972)	98.11
235791	1.48	0.82	0.47	0.67	Synergistetes ; Synergistaceae	*Aminobacterium colombiense* (NR_074624)	97.73
661359	0.03	1.14	0.30	0.29	Synergistetes ; Synergistaceae	Cloacibacillus porcorum (NR_109636)	90.98
260742	1.28	2.32	0.89	0.46	Synergistetes ; Synergistaceae	*Cloacibacillus porcorum* (NR_109636)	90.98
555847	0.02	1.12	0.36	0.32	Synergistetes ; Synergistaceae	*Cloacibacillus porcorum* (NR_109636)	90.6
777316	16.22	4.65	10.84	10.57	Thermotogae ; Petrotogaceae	*Defluviitoga tunisiensis* (NR_122085)	99.25
