# Supplementary material for: Role of biochar in anaerobic microbiome enrichment and methane production enhancement during olive mill wastewater biomethanization
Source: Front Bioeng Biotechnol. 2023 Jan 4;10:1100533. doi: 10.3389/fbioe.2022.1100533 (PMC9846136; doi:10.3389/fbioe.2022.1100533)
Supplement: Supplementary file 2 [file Table2.pdf]

Table S2: Phylogenetic affiliation of archaeal OTUs obtained from Illumina Miseq sequencing analysis in control and biochar supplemented batches targeting the 16S rRNA gene

Sequences per sample (%)				Closet cultivated relative from NCBI nucleotide database		
S0	S1	L1	T1	Taxonomy (phylum/family)	Species (accession number)	Similarity (%)
0.00	0.01	0.01	0.01	Crenarchaeota ; Nitrososphaeraceae	*Nitrososphaera viennensis* (NR_134097)	94.34%
0.05	0.14	0.03	0.03	Euryarchaeota ; Methanobacteriaceae	*Methanobacterium beijingense* (NR_028202)	97.37%
0.04	0.09	0.02	0.02	Euryarchaeota ; Methanobacteriaceae	*Methanobacterium beijingense* (NR_028202)	96.62%
0.00	0.01	0.00	0.02	Euryarchaeota ; Methanobacteriaceae	*Methanobacterium beijingense* (NR_028202)	98.87%
0.02	0.05	0.01	0.02	Euryarchaeota ; Methanobacteriaceae	*Methanobacterium aggregans* (NR_135896)	98.12%
0.01	0.02	0.01	0.01	Euryarchaeota ; Methanobacteriaceae	*Methanobacterium beijingense* (NR_028202)	96.24%
0.01	0.00	0.01	0.01	Euryarchaeota ; Methanocorpusculaceae	*Methanocorpusculum aggregans* (NR_117749)	99.25%
0.01	0.00	0.02	0.01	Euryarchaeota ; Methanocorpusculaceae	*Methanocorpusculum aggregans* (NR_117749)	98.87%
0.09	0.01	0.01	0.01	Euryarchaeota ; Methanomicrobiaceae	*Methanoculleus receptaculi* (NR_043961)	99.62%
0.29	0.01	0.04	0.04	Euryarchaeota ; Methanomicrobiaceae	*Methanoculleus receptaculi* (NR_043961)	98.87%
0.07	0.00	0.02	0.01	Euryarchaeota ; Methanomicrobiaceae	*Methanoculleus receptaculi* (NR_043961)	99.25%
0.03	0.00	0.01	0.01	Euryarchaeota ; Methanomicrobiaceae	*Methanoculleus receptaculi* (NR_043961)	99.61%
0.00	0.00	0.00	0.00	Euryarchaeota ; Methanomicrobiaceae	*Methanofollis ethanolicus* (NR_041669)	97.35%
0.01	0.02	0.01	0.02	Euryarchaeota ; Methanotrichaceae	*Methanothrix harundinacea (NR_043203)*	96.62%
0.05	0.08	0.07	0.10	Euryarchaeota ; Methanotrichaceae	Methanothrix harundinacea (NR_043203)	96.99%
0.05	0.07	0.05	0.06	Euryarchaeota ; Methanotrichaceae	Methanothrix soehngenii (NR_102903)	98.87%
0.00	0.02	0.03	0.08	Euryarchaeota ; Methanosarcinaceae	*Methanosarcina barkeri* (NR_118371)	99.25%
0.49	0.02	0.09	0.37	Euryarchaeota ; Methanomassiliicoccaceae	*Methanomassiliicoccus luminyensisB10* (NR_118098)	93.58%
0.02	0.00	0.01	0.02	Euryarchaeota ; Methanomassiliicoccaceae	*Methanomassiliicoccus luminyensis* B10 (NR_118098)	93.58%
0.01	0.00	0.02	0.01	Euryarchaeota ; Methanomassiliicoccaceae	*Methanomassiliicoccus luminyensis* B10 (NR_118098)	90.94%
0.01	0.00	0.01	0.01	Euryarchaeota ; Methanomassiliicoccaceae	*Methanomassiliicoccus luminyensis* B10 (NR_118098)	90.60%
0.04	0.01	0.03	0.08	Euryarchaeota ; Methanomassiliicoccaceae	*Methanomassiliicoccus luminyensis* B10 (NR_118098)	89.81%
0.37	0.01	0.03	0.11	Euryarchaeota ; Methanomassiliicoccaceae	*Methanomassiliicoccus luminyensis* B10 (NR_118098)	89.85%
0.05	0.00	0.01	0.01	Euryarchaeota ; Methanomassiliicoccaceae	*Methanomassiliicoccus luminyensis* B10 (NR_118098)	89.85%
0.06	0.01	0.03	0.02	Euryarchaeota ; Methanomassiliicoccaceae	*Methanomassiliicoccus luminyensis* B10 (NR_118098)	89.85%
0.03	0.01	0.02	0.05	Euryarchaeota ; Methanomicrobiaceae	*Methanomicrobium mobile* (NR_044726)	76.52%
0.01	0.00	0.01	0.01	Euryarchaeota ; Methanomassiliicoccaceae	*Methanomassiliicoccus luminyensis* B10 (NR_118098)	92.08%
