# Supplementary figures and images for: Role of biochar in anaerobic microbiome enrichment and methane production enhancement during olive mill wastewater biomethanization
Source: Front Bioeng Biotechnol. 2023 Jan 4;10:1100533. doi: 10.3389/fbioe.2022.1100533 (PMC9846136; doi:10.3389/fbioe.2022.1100533)

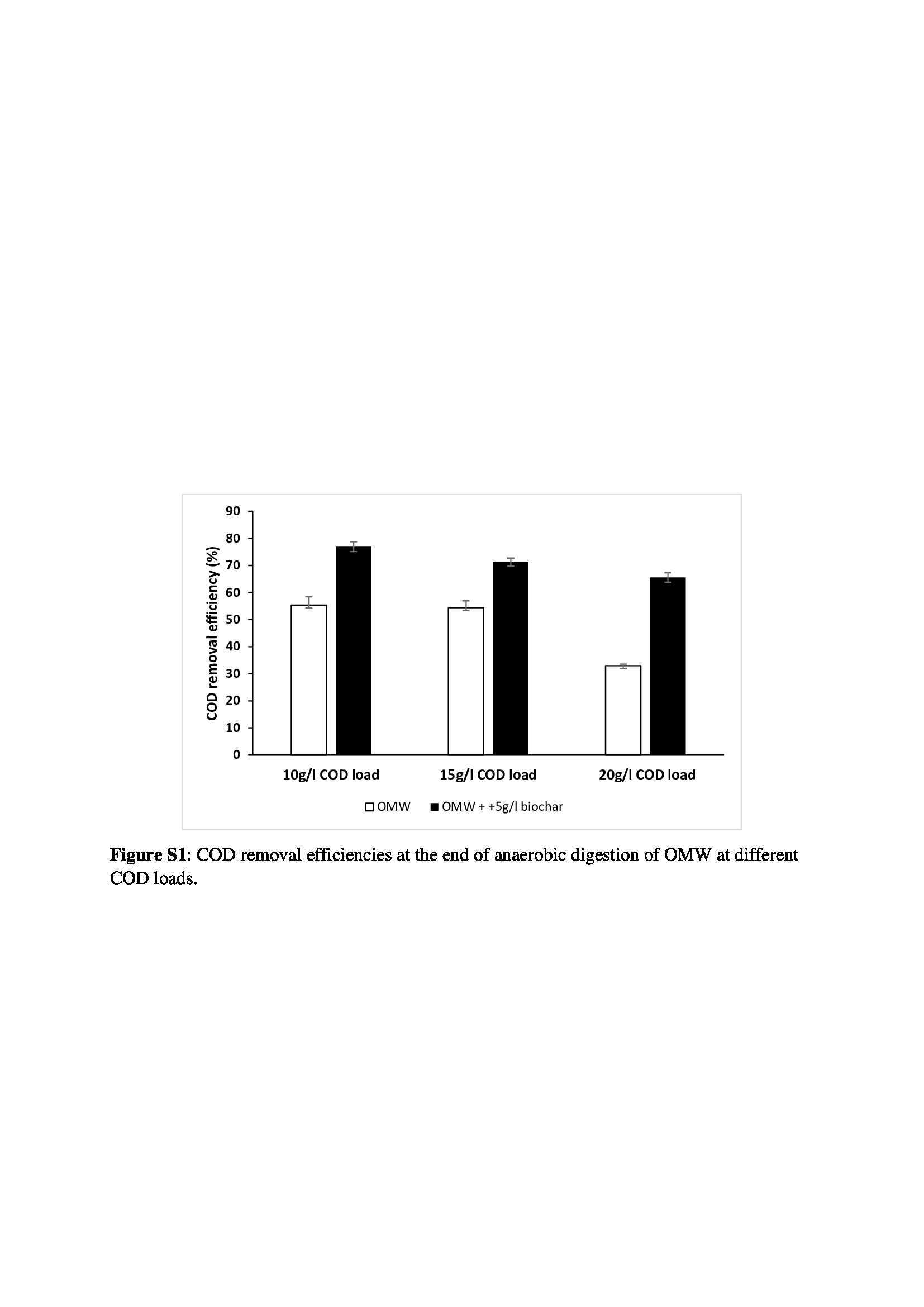

Supplement: Supplementary file 3 [file Image1.jpeg]
